# Impact of an Intervention Aimed at Improving Sleep Quality in Hospitalized Children

**DOI:** 10.3390/children11070848

**Published:** 2024-07-12

**Authors:** Carolina Lechosa-Muñiz, Laura Ruiz-Azcona, Elena Pérez Belmonte, María Paz-Zulueta, María Jesús Cabero-Pérez

**Affiliations:** 1 Departamento de Enfermería, Universidad de Cantabria, Avda. Valdecilla s/n, 39008 Santander, Spain; 2 Pediatrics Section, Hospital Universitario Marqués de Valdecilla, Avda. Valdecilla s/n, 39008 Santander, Spain; 3 IDIVAL—Instituto de Investigación Sanitaria Valdecilla, Avda. Cardenal Herrera Oria s/n, 39011 Santander, Spain; 4 Global Health Research Group, Universidad de Cantabria, Avda. Valdecilla s/n, 39008 Santander, Spain; 5 Departamento de Ciencias Médicas y Quirúrgicas, Universidad de Cantabria, Avda. Cardenal Herrera Oria s/n, 39011 Santander, Spain

**Keywords:** pediatrics, sleep, hospitalization, sleep deprivation, sleep hygiene

## Abstract

Background: Hospitalized children need adequate sleep to favor early recovery. Methods: To study the sleep pattern of children admitted to a pediatric inpatient unit, a cross-sectional study was carried out at a reference hospital in northern Spain. The main study variables were medical specialty of admission, sleep-inducing treatment, hours of sleep at home and during admission, number of nocturnal awakenings, and reasons for awakening. Differences in the hours of sleep and nighttime awakenings between the initial period and at six months were calculated using the Student’s *t*-test. Results: We included 100 baseline patients and 100 post-intervention patients. Up to 4% of the baseline sample and 3% of the six-month sample had been prescribed a sleep-promoting drug. Regarding awakenings, 79% of the children in the baseline sample suffered awakenings, with a mean of 1.98 awakenings (range 1–13). At six months, the percentage of children who experienced awakenings decreased by 17%, with a mean of 1.34 (range 1–5). In the baseline sample, 48% were caused by nursing care, decreasing to 34% after the intervention. Conclusions: An educational intervention with the implementation of targeted evidence-based practices is a useful measure for improving the sleep pattern by decreasing the number of awakenings.

## 1. Introduction

Sleep is a necessary neurophysiological process for brain development since it is involved in multiple cognitive processes. Sleep patterns are conditioned by intrinsic neurological circuits, fulfilling a restorative function at a physiological level, with variations to both sleep patterns and sleep needs according to age [1].

Sleep and rest needs in children and adolescents are age dependent. This is especially true in infants, with a changing sleep pattern that evolves throughout childhood for optimal neurological and physical development [2].

According to the American Academy of Sleep Medicine (AASM) and the American Academy of Pediatrics (AAP) [3], as well as the World Health Organization (WHO), the average hours of sleep during the first year of life should be 12 to 16 h per day. As children grow older, this time pattern decreases to 11 to 14 h in children aged 1 to 2 years, 10 to 13 h in preschoolers, 9 to 12 h in school children, and 8 to 10 h in adolescents under 18 years of age [4]. 

In healthy children and adolescents, the absence and disruption of sleep can delay the achievement of developmental milestones, disrupt academic performance, and contribute to the development or aggravation of mental illnesses and behavioral disorders, especially in adolescents [5,6]. 

It is important to note that sleep disorders are very common in children and adolescents [7]. About 20–30% of the child population has a sleep-related problem that requires intervention, as sleep-related problems interfere with the physical, cognitive, emotional, and social development of children. In addition, if a sleep-related problem begins, 25–84% tend to persist in early childhood for up to three years [8]. 

Hospitalization has a detrimental impact on the sleep quality of children, as a stressor that exceeds their adaptive capacity, negatively influencing their health [9]. Although the literature on sleep in pediatric patients is less extensive than in adults, several studies outline the magnitude of the problem [10,11,12,13,14]. Generally, during hospitalization, the alterations described in different studies are related with alterations in sleep–wake cycles and sleep fragmentation, i.e., frequent awakenings, rather than with a decrease or deprivation in the number of hours of sleep [11,14,15]. 

Poor sleep during hospital admission may have implications for the child’s adjustment at home after discharge. It is common for children who have experienced sleep problems during their hospital stay to experience behavioral changes at home, including generalized anxiety, apathy, and withdrawal [16].

Several factors can alter the sleep pattern during hospitalization, one of the most relevant is the hospital stay itself. However, certain external factors such as noise, light, room temperature, and interventions performed by healthcare personnel (monitoring of vital signs, administration of medication, invasive procedures, etc.) can also affect the sleep pattern during hospitalization [17], together with clinical factors depending on the patient’s pathology, such as pain, respiratory distress, recurrent coughing episodes, fever, frequent nocturnal urination, etc., which may affect and have an impact on the quality of children’s sleep during a hospital stay [18,19,20,21]. 

Hospitalized children need adequate sleep to promote early recovery, and it is necessary to implement interventions aimed at preserving and improving the quality of sleep and rest in hospitalized pediatric patients [22]. Behavioral interventions are one of the possible strategies to promote sleep during hospitalization. These include relaxation techniques to reduce hospital anxiety, combined with sleep education interventions aimed at families [23]. Currently, there is evidence that non-pharmacological interventions can reduce sleep disturbances, both inside and outside the hospital. Therefore, it is essential to raise awareness among healthcare professionals and families about the need to provide appropriate environments to promote sleep and rest in children [14,24].

This study is very relevant, because despite the large number of studies on sleep in children hospitalized, most of the latter reflect the descriptive part of the problems associated with shortening sleep or awakenings during hospitalization. It is necessary to explore sleep patterns in hospitalized children and to analyze the external factors associated with sleep disturbances.

The aim of this study was to study the sleep pattern of children admitted to a pediatric inpatient unit and the changes in this pattern after an educational intervention and after the implementation of evidence-based practices aimed at improving the sleep pattern in relation to the number of hours of sleep and awakenings.

## 2. Methods

### 2.1. Study Design and Participants

A cross-sectional study was carried out at the “Marqués de Valdecilla” University Hospital (HUMV), a reference hospital in northern Spain and a third level hospital of the National Health System, concretely, at the pediatric inpatient unit of this hospital, where 1500 children are admitted each year for an average stay of 3.08 days. This unit has 24 single rooms and an occupancy rate of 53% (2020). It has a multiprofessional team with three assistant pediatricians, two resident physicians, twelve nurses, nine auxiliary nurses, and a nursing supervisor. This unit admits children aged from one month to 16 years old with different pediatric pathologies in the areas of neurosurgery, maxillofacial, traumatology, pediatric surgery, and psychiatry.

The study participants were children aged 2–16 years admitted to the pediatric inpatient unit who were physically and mentally healthy without previous sleep disorders. The exclusion criteria were chronic systemic disease, patients with infantile cerebral palsy, or sedative or hypnotic drug use. Also, children aged 0–2 years were excluded from the sample because in the first 24 months of life children do not have an established sleep pattern [25].

All the families of the children who met the inclusion criteria during their hospital stay were informed of the study and were asked for their written informed consent for inclusion in the study. In addition, children were also asked for informed consent, adapted according to the patient’s age (young children or adolescents).

The main study variables were age, sex, medical specialty of admission, the prescription of any sleep-inducing drugs, hours of sleep at home and during admission, number of nocturnal awakenings, and reasons for awakening.

To assess the sleep pattern at home (prior to hospitalization), the Spanish version of the BEARS Questionnaire was used [26]. This questionnaire assesses five main aspects of sleep: bedtime problems, excessive daytime sleepiness, nighttime awakenings, regularity and duration of sleep, and snoring. This questionnaire is applied in three age brackets: 2 to 5 years, 6 to 12 years, and 13 to 18 years, with questions addressed to parents and older children [27].

After establishing the history of sleep disorders and once the child was hospitalized, the sleep pattern was monitored for 24 h by means of a sleep diary, which were filled out by the parents of the patients who remained in the room with the child. This consisted of five questions that were asked to parents or guardians: time of onset of nighttime sleep, number and time of nighttime awakenings, reasons for awakenings, and time of morning awakening.

### 2.2. Implementation of Evidence-Based Practices

To evaluate the effectiveness of the educational intervention and the application of evidence-based practices, the following steps were followed between the first sample (100 baseline patients) and the second sample (100 post-intervention patients):

- A literature review was carried out to delve into the interventions with scientific evidence aimed at promoting sleep and rest in hospitalized children. We found that behavioral-educational interventions for bedtime problems (graduated extinction or bedtime fading/positive routines for example) and preventive parent education have received support across different studies [3,16,23,28,29,30,31].
- A comprehensive protocol was drawn up for pediatric patient sleep care in the different phases. To this end, training sessions were held, and the protocol was disseminated to the entire healthcare team. During pre-admission, we assessed whether the patient slept in a crib or bed. On admission, the nursing assessment was carried out following the 14 Basic Needs model of Virginia Henderson [32]. During the night shift, the healthcare personnel provided an optimal environment for the reconciliation of the hospitalized child’s sleep with interventions such as ideal temperature, dark and quiet room, and grouping of care and schedules (Table S1).
- A behavioral-educational intervention was carried out that included sleep education for parents and children and training in the use of a diaphragmatic breathing exercises, for which a video was recorded with Koeppen’s relaxation techniques for children under 10 years of age and another video with Jacobson’s relaxation techniques for children over 10 years of age [33,34]. QR codes were generated for access to these videos, which were provided to all families. Upon admission to the unit, parents were explained the possibility of using the QR code to facilitate their children’s conciliation of sleep. Children were encouraged to practice the technique and use it at least once a day, after nighttime wakings, or if experiencing anxiety related to medical or nursing care.

### 2.3. Statistical Analysis

The sample size was calculated with the EPIDAT version 3.1 statistical program based on the study population (total admissions in 2020 in the pediatric hospitalization ward) (N = 1500), by calculating proportions. Using a 95% confidence level with an expected proportion of 50% due to the lack of official data on this proportion, the maximum sample size was chosen with a maximum estimation error of 5%. A sample size of 95 was obtained to achieve the main objective, to which 5% was added for possible losses due to unwillingness to participate, resulting in a sample size of 100 baseline patients and 100 post-intervention patients. A one-month recruitment period was estimated until the sample size was reached and the evaluation of the educational intervention and the implementation of evidence-based practices was established at six months [35].

The data were collected by the nurses. With the information obtained, a descriptive analysis was performed and the total daily sleep time at home and during admission was calculated, as well as the number of nocturnal awakenings and the elements that motivated the sleep interruptions at the hospital. Differences in the number of hours of sleep and the number of nocturnal awakenings between the initial period and at six months were determined using the Student’s *t*-test. For the significance level of the tests, *p*-values < 0.05 and <0.01 were set as thresholds. All statistical analyses were performed with the the SPSS 20.0 package.

## 3. Results

Table 1 presents the characteristics of the study participants in terms of age, sex, medical specialty, and sleep-promoting pharmacological treatment of the baseline sample and in the 6-month post-intervention sample.

The mean age of the patients in the baseline sample was 8.07 years (SD = 4.52), whereas in the sample at six months, the mean age was 9.39 years (SD = 4.23), *p* = 0.034. As for sex, 46% of the baseline sample were women and 54% men, whereas at six months, 42% were women and 58% men, although this difference was not statistically significant. 

The patients were admitted for various pathologies; 64% of the patients in the baseline sample were related to the medical specialty of pediatrics, 18% to pediatric surgery, 11% to psychiatry, 5% to traumatology, and 2% to maxillofacial surgery. The sample at six months post-treatment showed a similar distribution: 65% were related to pediatrics, 21% to pediatric surgery, 8% to psychiatry, 3% to traumatology, 2% to neurosurgery, and 1% to maxillofacial surgery.

Concerning pharmacological treatments, only 4% of the baseline sample and 3% of the sample at six months had been prescribed a sleep-promoting drug.

Table 2 presents the results on the sleep characteristics of the pediatric patients included in the study.

The mean number of hours of sleep at home in the baseline sample was 9.25 h [1.07], whereas at six months it was 9.09 h [0.93]. This difference was not statistically significant. Regarding awakenings, 25% of the baseline sample suffered awakenings at home, compared to 20% in the sample at six months.

In the hospital, the mean number of hours of sleep in the baseline sample was 9.09 h [1.18] and 9.28 h [1.02] at six months, without reaching statistical significance. Regarding awakenings during admission, in the baseline sample 79% of the children suffered awakenings, with a mean of 1.98 awakenings (range 1 to 13). At six months, the percentage of children who suffered awakenings decreased by 17%, with a mean of 1.34 awakenings, (range 1 to 5).

Regarding the reasons for awakening, in the baseline sample, 48% were caused by nursing care, 46% by reasons derived from the pathology that caused the hospital admission (pain, respiratory problems, anxiety, urination, etc.), 13% by noise, 6% by room temperature, and 5% by light. At six months, 37% of the awakenings were caused by reasons derived from the pathology that caused the hospital admission, 34% by nursing care, 13% by noise, 3% by room temperature, and 3% by light. These results did not reach statistical significance.

The educational intervention was performed in 63% of patients in the sample at 6 months. The implementation of evidence-based practices (protocols and care plan) was carried out in the entire pediatric inpatient unit. 

## 4. Discussion

The analysis of the data obtained in our study shows that the number of hours of nighttime sleep (nine hours) is similar both at home and at hospital, prior to the educational intervention and the implementation of evidence-based practices.

Our results agree with the studies of Stickland et al., 2016 and Berger et al., 2021 [11,14], who highlight that during hospitalization sleep pattern disturbances are mostly due to the number of nocturnal awakenings, rather than a decrease in the number of hours of sleep. However, they differ from findings by Bevan et al., 2019 [15], who measured sleep quality and noise levels in pediatric wards and compared them to the home environment, finding that children slept one hour less in the hospital than at home. Similarly, Lee et al., 2017 [36] reported that, at hospital, pediatric patients slept fewer hours at night, and for some children, this meant a clinically significant decrease in total sleep time. However, these results are possibly due to the characteristics of the patients since they were pediatric cancer patients.

In relation to the number of awakenings, if we compare the home and the hospital, we find that in the hospital there are a greater number of awakenings. In the baseline sample, 25% suffered awakenings at home compared to 79% at hospital. This is mainly due to external environmental factors.

These results agree with the study by Vecchi, 2020 [37], who stated that hospital admission, regardless of the disease that caused it, does not favor restful and sufficient sleep. This author also identified a series of external (light and noise) and internal (procedures, drugs, and care) factors that interfere with sleep.

At six months, after the intervention, the number of awakenings was lower, from 1.98 awakenings on average with a range of 1 to 13 in the baseline sample and 1.34 at six months, with a range of 1 to 5.

The data analyzed in our study also show similarities with the study by Papaconstantinou et al., 2018 [16], who identified that children who had undergone a behavioral-educational intervention slept an average of 50 min longer at night than those in the control group.

Regarding the reason for awakenings, in the baseline sample, 48% were caused by nursing care, decreasing to 34% after the educational intervention and the implementation of evidence-based practices at six months. In the six-month sample, the highest percentage of awakenings was caused by reasons related to the pathology for which the patient was admitted (pain, respiratory problems, anxiety, urination, etc.).

When assessing the factors that interrupted sleep, these were in line with the study by Peirce et al., 2018 [19], who showed that interruptions by the nurse/physician were the most frequent reasons for sleep disturbances, according to the families. Recently, Farías-Fernández et al., 2021 [38] identified factors that interrupted the sleep of hospitalized pediatric patients, which included the placement of venoclysis and drainage systems, as well as the entry and exit of health staff to and from the rooms.

It is important to note that, in our sample, light and room temperature had a scarce influence on patient awakenings. In contrast, noise was a principal reason for awakenings. Crawford et al., 2019 [17] also showed that noise was one of the main causes of awakenings. Stickland et al., 2016 [11] in their study determined that noise and light were key factors that disturbed the sleep of hospitalized pediatric patients and their families. Similarly, in the systematic review by Lee et al., 2017 [36], noise, light, and staff entering the patient’s room were all associate ted with increased nighttime awakenings. 

### Limitations

In this study, given the characteristics of the admissions to our unit (mean length of stay of 3.08 days and mean of 125 admissions per month) and the various medical pathologies attended, it was not possible to assess the same children pre- and post-intervention. However, the characteristics of the children in both samples were similar, as was the sleep pattern.

## 5. Conclusions

Hospitalization alters sleep patterns, even in children who had no previous sleep disorder before hospitalization. It is necessary to explore sleep patterns in hospitalized children and to analyze the external factors that may disrupt sleep.

A nursing assessment during admission enables the establishment of a global care plan that provides an optimal environment for the reconciliation of the hospitalized child’s sleep, with interventions to maintain an ideal temperature, to ensure that the room remains dark and free of noise, and to try to group nursing care and schedules. 

An educational intervention and the implementation of targeted evidence-based practices are useful measures to improve the sleep pattern by decreasing the number of awakenings.

## Figures and Tables

**Table 1:** Characteristics of the patients included in the study.

	Baseline		6 Months		
	n = 100	%	n = 100	%	*p*-Value
Mean Age [SD]	8.07 [4.52]		9.39 [4.23]		0.034
<6 years	37	37%	23	23%	0.096
6–12 years	40	40%	48	48%	
>12 years	23	23%	29	29%	
Gender					
Woman	46	46%	42	42%	0.569
Male	54	54%	58	58%	
Medical Specialty					
Neurosurgery	0	0	2	2%	0.617
Maxillofacial	2	2%	1	1%	
Traumatology	5	5%	3	3%	
Pediatric surgery	18	18%	21	21%	
Psychiatry	11	11%	8	8%	
Pediatrics	64	64%	65	65%	
Sleep promoting drugs					
No	96	96%	97	97%	0.700
Yes	4	4%	3	3%	

**Table 2:** Sleep characteristics of the pediatric patient at home vs. hospitalization.

	Baseline		6 Months		
	n = 100	%	n = 100	%	*p*-Value
At home:					
Hours of sleep [SD]	9.25 [1.07]		9.09 [0.93]		0.249
Awakenings					
No	74	74%	79	79%	
Yes	25	25%	20	20%	0.396
At the hospital:					
Hours of sleep [SD]	9.09 [1.18]		9.28 [1.02]		0.212
Awakenings					
No	21	21%	38	38%	0.320
Yes	79	79%	62	62%	
Number of awakenings [SD]	1.98 [2.09]		1.34 [1.41]		0.012
(range: min, max)	1–13		1–5		
Reasons for awakening					
Noise	13	13%	13	13%	1.000
Light	5	5%	3	3%	0.470
Room temperature	6	6%	3	3%	0.306
Nursing care	48	48%	34	34%	0.044
Others	46	46%	37	37%	0.196
Educational intervention					
No	100	100%	37	37%	
Yes	0		63	63%	

## Data Availability

The data presented in this study are available on request from the corresponding author (L.R.-A.) due to privacy.

## Supplementary Materials

The following supporting information can be downloaded at: https://www.mdpi.com/article/10.3390/children11070848/s1, Table S1: Care plan using NANDA, NOC and NIC taxonomy.
