# Supplementary material for: Impact of an Intervention Aimed at Improving Sleep Quality in Hospitalized Children
Source: Children (Basel). 2024 Jul 12;11(7):848. doi: 10.3390/children11070848 (PMC11276261; doi:10.3390/children11070848)
Supplement: Supplementary file 1 [file children-11-00848-s001.zip › children-3094701-supplementary.pdf]

Table S1. Care plan using NANDA, NOC and NIC taxonomy.

**00198 SLEEP PATTERN DISORDER**<br>Definition: awakening due to external factors for a limited time period.	
RELATED FACTORS	Environmental disturbances.
DEFINING CHARACTERISTICS	Difficulty falling asleep.<br>Difficulty staying asleep.<br>Non-restorative sleep-wake cycle.<br>Expresses dissatisfaction with sleep.<br>Expresses tiredness.
**NOC**	**NIC**
**0004 Sleep**<br><br>Indicators:<br><br>• [401] Hours of sleep.<br>• [419] Comfortable bed.<br>• [420] Comfortable room temperature.<br><br>SCALE 1:<br>1. Severely compromised.<br>2. Substantially compromised.<br>3. Moderately compromised.<br>4. Mildly compromised.<br>5. Uncompromised.<br><br>Indicators:<br><br>• [406] Interrupted sleep.<br>• [421] Difficulty falling asleep.<br>• [422] Nightmares.	**1850 Improving sleep**<br><br>Activities:<br><br>✓ Determine the patient's sleep/wake pattern.<br>✓ Include the patient's regular sleep/wake cycle in care planning.<br>✓ Observe/record the patient's sleep pattern and number of hours of sleep.<br>✓ Check the patient's sleep pattern and note physical (sleep apnea, obstructed airway, pain/discomfort, and urinary frequency) and/or psychological (fear or anxiety) circumstances that disrupt sleep.<br>✓ Adjust the environment (light, noise, temperature, mattress, and bed) to promote sleep.<br>✓ Help the patient avoid foods and beverages that interfere with sleep at bedtime.<br>✓ Arrange for naps during the day, if indicated, to meet sleep needs.<br>✓ Adjust the medication administration schedule to support the patient's sleep/wake cycle.<br>✓ Regulate environmental stimuli to maintain normal day-night cycles.<br>✓ Discuss techniques to promote sleep with the patient and family.<br><br>**6482 Environmental management: comfort**<br><br>Activities:<br><br>✓ Determine patient and family goals for environmental manipulation and optimal comfort.<br>✓ Prepare the patient and family for the transition by warmly welcoming them to the new environment.

SCALE 14:<br>1. Severe.<br>2. Substantial.<br>3. Moderate.<br>4. Mild.<br>5. None.<br><br>**2009 Comfort level: environment**<br><br>Indicators:<br>• [200902] Room temperature.<br>• [200903] Favorable sleep environment.<br>• [200909] Room lighting.<br>• [200912] Comfortable bed.<br>• [200913] Comfortable furnishings.<br>• [200916] Noise control.<br><br>SCALE 1<br>1. Severely compromised.<br>2. Substantially compromised.<br>3. Moderately compromised.<br>4. Slightly compromised.<br>5. Uncompromised.	✓ Immediately respond to calls from the buzzer, which should always be within the patient's reach.<br>✓ Avoid unnecessary interruptions and allow for rest periods.<br>✓ Create a calm and supportive environment.<br>✓ Provide a clean and safe environment.<br>✓ Determine sources of discomfort, such as wet dressings, probe position, constrictive dressings, wrinkled bedding, and environmental irritants.<br>✓ Set the room temperature that is most comfortable for the person, if possible.<br>✓ Provide or remove blankets to promote temperature comfort, if applicable.<br>✓ Avoid unnecessary exposure, drafts, excessive heating or cold.<br>✓ Adjust lighting to suit the person's activities, avoiding direct light in the eyes.<br>✓ Facilitate hygiene measures to maintain the person's comfort.<br>✓ Position the patient in a way that facilitates comfort (using principles of body alignment, pillow support, joint support during movement, splints for the painful part of the body).<br>✓ Monitor the skin, especially body prominences, for signs of pressure or irritation.
